# Histone Deacetylase Inhibition Regulates Lipid Homeostasis in a Mouse Model of Amyotrophic Lateral Sclerosis

**DOI:** 10.3390/ijms222011224

**Published:** 2021-10-18

**Authors:** Thibaut Burg, Elisabeth Rossaert, Matthieu Moisse, Philip Van Damme, Ludo Van Den Bosch

**Affiliations:** 1 Department of Neurosciences, Experimental Neurology, Leuven Brain Institute, KU Leuven—University of Leuven, 3000 Leuven, Belgium; thibaut.burg@kuleuven.be (T.B.); elisabeth.rossaert@kuleuven.be (E.R.); matthieu.moisse@genadvice.com (M.M.); philip.vandamme@kuleuven.be (P.V.D.); 2 Center for Brain & Disease Research, Laboratory of Neurobiology, VIB, 3000 Leuven, Belgium; 3 Department of Neurology, University Hospitals Leuven, 3000 Leuven, Belgium

**Keywords:** amyotrophic lateral sclerosis, FUS, histone deacetylase, epigenetic, HDAC inhibitors, glycerophospholipids, lipidomic, metabolism

## Abstract

Amyotrophic lateral sclerosis (ALS) is an incurable and fatal neurodegenerative disorder of the motor system. While the etiology is still incompletely understood, defects in metabolism act as a major contributor to the disease progression. Recently, histone deacetylase (HDAC) inhibition using ACY-738 has been shown to restore metabolic alterations in the spinal cord of a FUS mouse model of ALS, which was accompanied by a beneficial effect on the motor phenotype and survival. In this study, we investigated the specific effects of HDAC inhibition on lipid metabolism using untargeted lipidomic analysis combined with transcriptomic analysis in the spinal cord of FUS mice. We discovered that symptomatic FUS mice recapitulate lipid alterations found in ALS patients and in the SOD1 mouse model. Glycerophospholipids, sphingolipids, and cholesterol esters were most affected. Strikingly, HDAC inhibition mitigated lipid homeostasis defects by selectively targeting glycerophospholipid metabolism and reducing cholesteryl esters accumulation. Therefore, our data suggest that HDAC inhibition is a potential new therapeutic strategy to modulate lipid metabolism defects in ALS and potentially other neurodegenerative diseases.

## 1. Introduction

Amyotrophic lateral sclerosis (ALS) is an adult-onset neurodegenerative disease primarily affecting the motor system. Due to the loss of upper and lower motor neurons, patients suffer from progressive muscle weakness and paralysis, typically leading to death within 2 to 5 years after the initial diagnosis [1]. Approximately 90% of cases occur sporadically, while the remaining 10% of ALS patients have a familial history characterized by autosomal dominant inheritance. Over the past decades, more than 30 genes have been identified that confer a major risk for ALS, and at least 120 genes were linked to the disease. The most common genetic causes of familial ALS, explaining about 70% of all familial ALS cases in the Western world, are missense mutations in the *superoxide dismutase 1* (*SOD1*), *TAR DNA binding protein 43* (*TARDBP*), and *fused in sarcoma* (*FUS*) genes as well as hexanucleotide expansions in the *chromosome 9 open reading frame 72* (*C9orf72*) gene [1]. These genetic discoveries have significantly contributed to the development of cellular and animal models and continue to develop our understanding of the disease. Nevertheless, the multifactorial pathological mechanisms are still incompletely understood, and the translation of treatments to the clinic has been poor. Therefore, better therapeutic strategies are urgently needed.

Recent evidence shows that epigenetic factors can modulate the phenotypic expression of ALS pathology and might explain part of its etiology [2,3]. One of the epigenetic marks associated with ALS is histone acetylation, which is a major regulator of gene expression [4]. In several models of the disease, global histone acetylation levels are decreased, mainly due to the dysregulation of histone deacetylase (HDAC) expression and activity [5,6,7,8,9,10]. These enzymes are grouped into four classes depending on their sequence and structural homology. Class I includes HDAC1-3, 8. Class II is divided into class IIa, including HDAC4, 5, 7, 9, and class IIb, including HDAC6, 10. Class III includes SIRT1-7. Class IV is represented only by HDAC11 [11]. While the activity of some HDACs contributes to disease onset and progression, other isoforms play beneficial roles. Consequently, the selective modulation of the activity of specific HDAC isoforms has the potential to modify the disease course of ALS [11]. In this context, we and others provided substantial evidence for the use of selective HDAC inhibitors in ALS [6,9,12,13,14,15,16,17]. Notably, we recently tested the efficacy of ACY-738, a potent and tolerable HDAC inhibitor with a selectivity profile toward HDAC6 and class I HDACs and the ability to cross the blood-brain barrier [6,12,18,19,20]. In vitro, we demonstrated using motor neurons derived from *FUS*-ALS patients that ACY-738 rescued axonal transport defects [12]. In vivo, we showed that ACY-738 treatment restored histone acetylation, slowed down disease progression and significantly expanded the lifespan of transgenic FUS mice, characterized by an aggressive ALS-like phenotype [6]. These homozygous transgenic FUS mice (*FUS^+/+^*) express wild-type human FUS under the control of the mouse prion promoter (PrP) [21]. Expression of the transgene in the nervous system leads to an early and progressive weight loss and motor performances impairment from 30 days of age and finally hindlimb paralysis at end-stage and death around 60 days [6].

Remarkably, FUS mice showed significant metabolic abnormalities in the spinal cord both at presymptomatic and symptomatic stages. Restoration of these defects by ACY-738 treatment correlated with the amelioration of the disease phenotype and demonstrated the link between epigenetic regulation and metabolism [6]. Among these metabolic defects, dysregulation of lipid homeostasis appeared as an early phenomenon [6]. These results align with growing evidence demonstrating that lipid metabolism dysfunction is not just a consequence of disease progression but rather plays a causal role [22,23,24]. Dysfunctions of the lipid metabolism are observed in the central nervous system, blood, and muscles of ALS patients and different mouse models, often long before the appearance of motor symptoms [25]. Lipidomic analyses on these tissues showed alterations of numerous lipid classes such as sphingolipids, glycerophospholipids, cholesterols, and free fatty acids [26,27,28,29,30,31,32]. In ALS patients, lipid-related biological parameters are strong predictors of survival [33,34,35,36]. Recently, several compounds aimed at modulating lipid metabolism have been shown to ameliorate motor performance and/or increase the lifespan of preclinical ALS mouse models [26,31,37]. Altogether, these studies indicate that targeting lipid metabolism defects is a rational therapeutic strategy for ALS.

In this context, we aimed to characterize the alterations of lipid homeostasis in the spinal cord of presymptomatic and symptomatic FUS mice and investigated the effect of HDAC inhibition. Untargeted lipidomic analysis showed that the lipid metabolism is altered in symptomatic *FUS* mice, with a significant dysregulation of glycerophospholipids, sphingolipids, and cholesteryl esters. Strikingly, ACY-738 treatment significantly restored lipid homeostasis, specifically targeting glycerophospholipids and cholesteryl esters. Moreover, transcriptome analysis showed that HDAC inhibition rescued the transcriptional activation of master transcription factors implicated in lipid metabolism and specifically restored gene expression alterations of key enzymes involved in the glycerophospholipid metabolism. Overall, our data confirm the dysregulation of lipid homeostasis in a novel FUS mouse model and provides new evidence that HDAC inhibition modulates lipid homeostasis. These data could severe as a stepping stone to the development of a new therapeutic strategy in ALS and other neurodegenerative diseases, such as Alzheimer’s disease and Parkinson’s disease, which share common lipid alterations.

## 2. Results

### 2.1. Lipidomic Analysis Reveals ALS Pathological Signature in the Spinal Cord of Symptomatic FUS Mice

**Figure 1:**
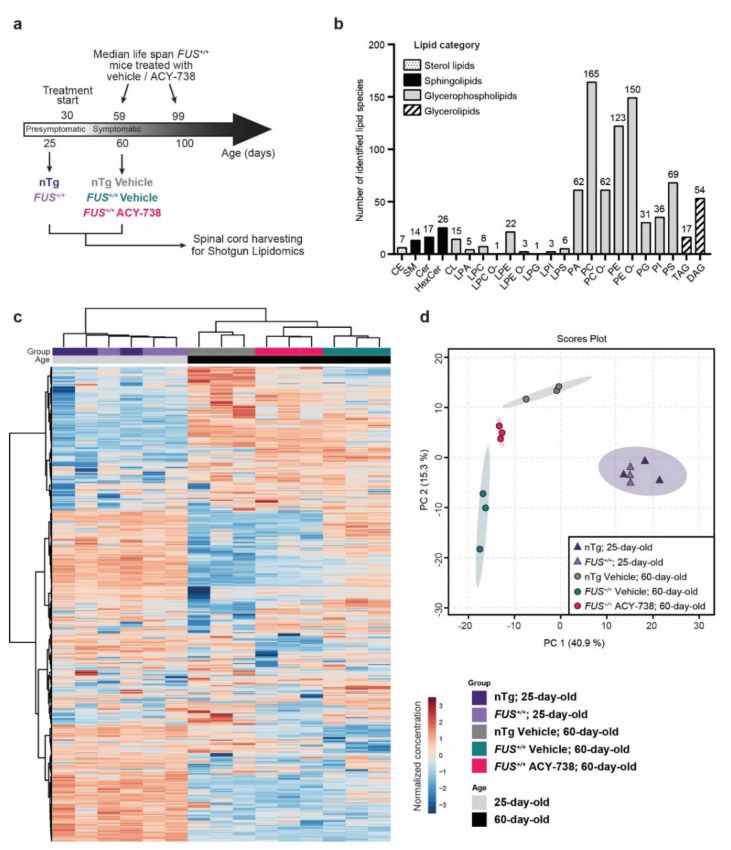
Whole spinal cord lipidome data show modifications of lipid homeostasis according to age, genotype, and treatment. (**a**) Schematic of the experimental design. *FUS^+/+^* mice develop motor symptoms after 30 days of age and have a median survival of 59 days [6,21]. Treatment with ACY-738, provided in crushed food mixed with water at a dose of 100 mg/kg, extends survival to 99 days and ameliorates disease phenotype [6]. Whole spinal cords were collected for shotgun lipidomics on presymptomatic (25-day-old) nTg and *FUS^+/+^* mice and symptomatic (60-day-old) vehicle-treated nTg and vehicle- or ACY-738-treated *FUS^+/+^* mice. (**b**) Number of identified lipid species per lipid class. Lipids classes are regrouped into four main categories. Abbreviations of the lipid classes are defined in the Supplementary Information File. (**c**) Heatmap representing all samples and color-coded normalized concentrations of all individual lipid species. The colors vary from deep blue to reddish-brown to indicate values below and above the mean normalized concentrations for a given lipid species, respectively. Cluster analysis was performed to display samples in columns and lipid species in rows. (**d**) Principal component analysis (PCA) scores plot of the whole lipidome data (15 samples; 538 lipid species).

To investigate how human *FUS* can affect lipid concentrations in the mouse spinal cord and how HDAC inhibition can modulate their homeostasis, we performed shotgun lipidomics. Lipids were extracted from the spinal cord of non-treated 25-day-old (presymptomatic) *FUS^+/+^* mice and vehicle- or ACY-738-treated 60-day-old (symptomatic) *FUS^+/+^* mice and their age-matched non-transgenic controls (nTg). ACY-738, a potent HDAC6 and class I HDAC inhibitor that can cross the blood-brain barrier [6,12,18,19,20], was administrated daily starting at symptom onset (30 days), as previously described [6] (Figure 1a).

The lipidome analysis using MS/MS technologies along with lipid class-specific internal standards allowed us to obtain absolute quantification of 23 lipid classes and 897 lipid species in picomole (Figure 1b). To compare samples with different total lipid amounts, data were transformed to the percentage of moles (mol%) per sample for each lipid species. Following data processing and normalization in MetaboAnalyst (see Materials and Methods), we restricted the analysis to 533 lipids distributed among 22 lipid classes. The overall lipid class proportions are relatively conserved across the five groups of this study (Supplementary Figure S1). We found that phosphatidylcholine (PC), phosphatidylethanolamine (PE), their ether-linked species (PC O-; PE O-), and their lysospecies (LPC; LPE) represented ≈70% of the total lipid mass in the spinal cord of each sample (Supplementary Figure S1) and showed the highest diversity of individual lipid species, with 534 species detected (Figure 1b). Hexocylceramide (HexCer) represented the second most abundant lipid class, counting for approximatively 12% of the total lipid mass. The remaining 18% of lipids were constituted of phosphatidylserine (PS; 8%) and sphingomyelin (SM; 4%), among others (Figure 1b, Supplementary Figure S1).

To visualize variation and to highlight robust phenotypes, we conducted multivariate analysis on the whole normalized data set. Principal component analysis (PCA) and hierarchical clustering analysis (HCA), displayed on a two-dimensional score plot and a heatmap plot (Figure 1c,d), showed the segregation of 4 groups among the 15 samples analyzed. Samples of the same experimental group clustered together, indicating excellent technical and biological reproducibility. These two statistical methods showed that samples from both 25-day-old nTg controls and *FUS^+/+^* mice clustered in one group apart from all samples from the 60-day-old animals. Among samples of 60-day-old animals, vehicle-treated nTg controls and *FUS^+/+^* mice formed two separate groups. Interestingly, samples from 60-day-old ACY-738-treated *FUS^+/+^* mice clustered in between vehicle-treated nTg controls and *FUS^+/+^* mice samples, as a tight separated group (Figure 1c,d).

Collectively, these results indicate the robustness of our lipidomic data and show that the lipid composition of a mouse spinal cord differs with age. Furthermore, these data also demonstrate that the effects of human *FUS* overexpression on lipid homeostasis are only present in symptomatic 60-day-old *FUS^+/+^* mice and that ACY-738 treatment can significantly modify lipid metabolism.

### 2.2. HDAC Inhibition Mitigates Lipid Metabolism Alterations in the Spinal Cord of Symptomatic FUS Mice

**Figure 2:**
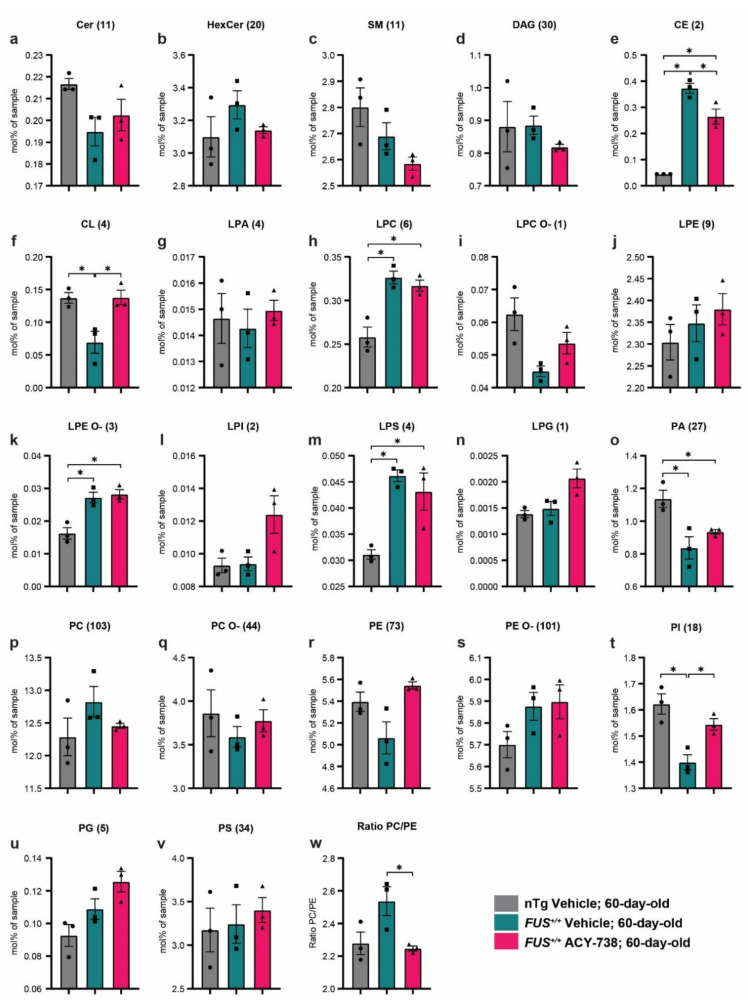
ACY-738 treatment mitigates specific lipid classes defects in 60-day-old mice *FUS^+/+^* mice (**a**–**v**) Differences in the concentration of all lipid classes analyzed in 60-day-old vehicle-treated nTg controls and vehicle- or ACY-738-treated *FUS^+/+^* mice. Each graph represents a distinct lipid class, and the number of lipids summed per class is indicated within brackets. (**w**) Ratio of the percentage of moles of sample from PC to PE in 60-day-old vehicle-treated nTg controls and vehicle- or ACY-738-treated *FUS^+/+^* mice. Data are in percentage of moles of the sample, presented as mean ± standard error of the mean (SEM). Statistical significance was calculated by 1-way ANOVA, followed by Tukey’s post-hoc test. * *p* < 0.05, FDR adjusted.

Next, we focused on 60-day-old mice to investigate the dysregulations of lipid metabolism caused by human *FUS* overexpression and how ACY-738 treatment impacted these alterations. First, we examined the concentration profile of lipid classes and looked for possible enrichment or diminution of whole specific lipid classes. Therefore, we transformed the absolute concentrations in picomoles to the percentage of moles per sample for each lipid, allowing us to directly compare samples with different total lipid amounts. To determine the concentration of each lipid class, we summed individual lipid species concentrations in mol% of samples. Among the 22 lipid classes analyzed, 6 presented significant alterations between the different groups (Figure 2). We observed significantly increased concentrations in vehicle- and ACY-738-treated *FUS^+/+^* mice compared to vehicle-treated nTg controls for three classes of lysophospholipids, specifically lyso-phosphatidylcholine (LPC), lysophosphatidylethanolamine-ether linked (LPE O-), and lyso-phosphatidylserine (LPS) (Figure 2h,k,m). Furthermore, phosphatidic acid (PA) concentrations were decreased in 60-day-old vehicle- and ACY-738-treated *FUS^+/+^* mice compared to vehicle-treated nTg controls (Figure 2o). Interestingly, the alterations of three other lipid classes in *FUS^+/+^* mice were totally or partially rescued by the ACY-738 treatment. Indeed, the decreased concentrations of cardiolipin (CL) and phosphatidylinositol (PI) in vehicle-treated *FUS^+/+^* mice were totally rescued in ACY-738-treated *FUS^+/+^* mice (Figure 2f,t). Moreover, the increased concentrations of cholesterol esters (CE) in vehicle-treated *FUS^+/+^* mice were partially rescued by ACY-738 treatment (Figure 2e).

A perfectly balanced ratio between the relative abundance of phosphatidylcholine (PC) and phosphatidylethanolamine (PE) is crucial for the maintenance of cell homeostasis, lipid droplets, mitochondria, and energy metabolism [38]. We observed a non-significant increase in the PC/PE ratio in 60-day-old vehicle-treated *FUS^+/+^* mice compared to vehicle-treated nTg controls. Interestingly, this ratio was significantly decreased in ACY-738-treated *FUS^+/+^* mice compared to vehicle-treated *FUS^+/+^* mice, reaching similar values to vehicle-treated nTg controls (Figure 2w).

Altogether, these results indicate that the overexpression of human *FUS* in mice impacts specific lipid classes, mostly glycerophospholipids. Furthermore, HDAC inhibition had beneficial effects on the modified concentrations of three lipid classes out of seven, namely, CE, CL, and PI.

### 2.3. HDAC Inhibition Targets Specific Individual Glycerophospholipid Species

**Figure 3:**
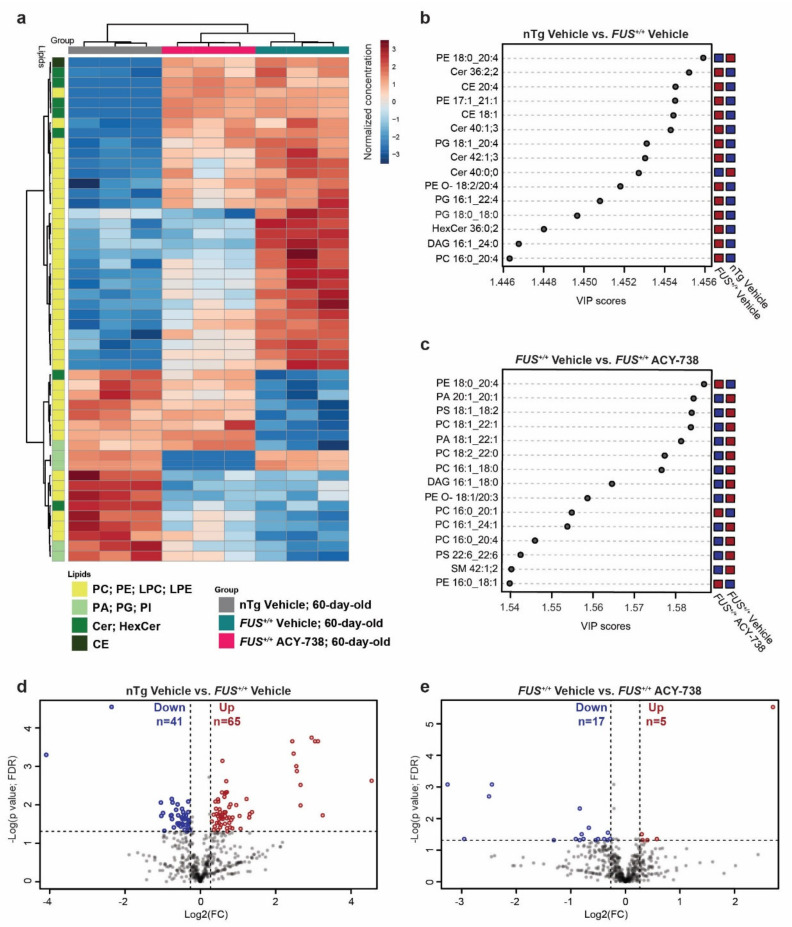
ACY-738 treatment ameliorates lipid homeostasis in the spinal cord of 60-day-old *FUS^+/+^* mice (**a**) Heatmap representing 60-day-old samples and color-coded normalized concentrations of the top 50 most significantly altered individual lipid species (1-way ANOVA, *p* < 0.05, FDR corrected). The colors vary from deep blue to reddish-brown to indicate, respectively, values below and above the mean normalized concentrations for a given lipid species. Cluster analysis was performed to display samples in columns and lipid species in rows. Selected lipid classes of interest are color-coded in shades of green (**b**,**c**) Top 15 discriminant lipidic features according to the variable importance on projection (VIP) score of 60-day-old vehicle-treated nTg controls and vehicle-treated *FUS^+/+^* mice (**b**) and vehicle-treated *FUS^+/+^* and ACY-738-treated *FUS^+/+^* mice (**c**). Red and blue colors indicate a high and a low normalized lipid concentration in each group, respectively. (**d**,**e**) Volcano plots depicting the log2 fold change (FC) of 538 lipid concentrations, comparison of vehicle-treated nTg controls to *FUS^+/+^* mice in (**d**) and vehicle-treated *FUS^+/+^* to ACY-738-treated *FUS^+/+^* mice in (**e**). A fold change of 1.2 and *p* > 0.05 (*t*-test; FDR corrected) were used to select significantly altered lipids, indicated as decreased in blue and increased in red. *n* = 3 per group.

The concentration of critical individual lipid species can vary without affecting the overall concentration of its lipid class. Moreover, the concentration of different individual lipid species can be both decreased and increased in one defined lipid class. Therefore, we next examined differences at the lipid molecular species level. To determine which individual lipid species contributed the most to the observed modification of lipid homeostasis, we performed both univariate and multivariate analyses (Figure 3; Supplementary Figures S2 and S3).

A heatmap displaying the 50 most dysregulated lipid species among the 3 experimental groups of 60-day-old mice showed enrichment of lipid species belonging to the glycerophospholipid category (42 out of 50). ACY-738 treatment was able to mitigate the increased or decreased concentrations of 24 lipid species out of 42. Interestingly, among these 24 lipid species, 22 were lipids with a choline or ethanolamine headgroup (Figure 3a). These observations were further confirmed by partial least squares discriminant analysis and the generation of a variable of importance in the projection score plot (VIP). This analysis allows estimating which lipids contributed most significantly to the differences observed between groups (Figure 3b,c). PC and PE lipids mainly contributed to the differences between 60-day-old vehicle-treated nTg controls and *FUS^+/+^* mice and between vehicle- and ACY-738-treated *FUS^+/+^* mice. Interestingly, CE and ceramides (Cer; HexCer) participated in the differences between vehicle-treated nTg controls and *FUS^+/+^* mice, but not between vehicle- and ACY-738-treated *FUS^+/+^* mice. These results indicate that ACY-738 treatment targets specific lipid classes, namely PC and PE. Next, we used volcano plots to display the most dysregulated lipids among the whole lipidome, with an adjusted *p* < 0.05 and a fold change FC > 1.2 (Figure 3d,e). We found 41 lipids with significantly decreased concentrations and 65 with significantly increased concentrations in vehicle-treated *FUS^+/+^* mice than nTg controls. Among these, 23 out of 41 and 46 out of 65 were lipids with a choline or ethanolamine headgroup. In ACY-738-treated *FUS^+/+^* mice compared to vehicle-treated *FUS^+/+^* mice, we observed 17 lipids with significantly decreased concentrations and 5 with significantly increased concentrations. Remarkably, we also observed the dysregulation and restoration by ACY-738 of several diacylglycerol (DAG) species, essential intermediates in the synthesis of glycerophospholipids. Fold change relative to nTg controls and concentrations in mol% of samples of these significantly dysregulated lipids are presented in the Supplementary Information (Supplementary Figures S2 and S3).

To conclude, we confirmed the dysregulation of individual lipid species belonging to the overall altered lipid classes (Figure 2). Again, lipids with a choline or ethanolamine headgroup were enriched with 9 out of 17 and 3 out of 5 lipids, respectively. These analyses also confirmed that CE and ceramides are dysregulated in mice overexpressing human *FUS* and are not the prime targets of HDAC inhibition. Different statistical analyses point out the predominant role of glycerophospholipids and specifically PC and PE in the pathogenic effect of human *FUS* overexpression and the beneficial effects of ACY-738.

### 2.4. HDAC Inhibition Partially Reinstates Fatty Acid Composition and Lipid Functional Properties

**Figure 4:**
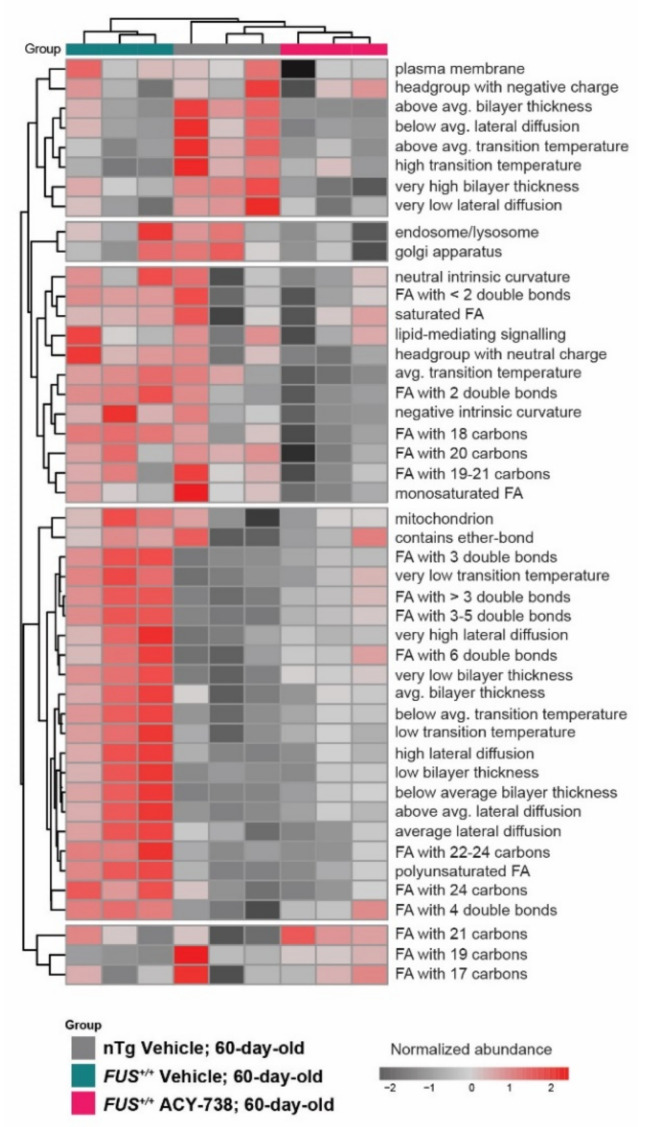
ACY-738 treatment partially reinstates enriched lipid ontology terms. Enrichment analysis of lipid ontology terms (LION) performed on normalized data. Each LION term represents a group of lipid species that share common properties. Heatmap representing clustered 60-day-old samples and color-coded enriched LION terms. Most significantly fluctuating LION terms for all the samples are displayed; red and gray colors indicate a high and a low enrichment. *n* = 3 per group.

To gain insight into the functional and biophysical properties of the dysregulated lipids, we took advantage of the web application LION/web to perform a lipid ontology enrichment analysis. This novel bioinformatic tool allowed us to search for enriched LION terms in our data set [39]. LION terms regroup lipids that share common functions, biophysical properties, fatty acids, and organelle associations. We selected the most fluctuating LION terms by assessing enrichment over a number of principal components. The selected LION terms plotted in the heatmap (Figure 4) showed that samples of each experimental group clustered together. LION terms enriched only in nTg control mice were related to plasma membrane properties with a high bilayer thickness, high transition temperature, and low lateral diffusion. Interestingly, a considerable proportion of LION terms was only enriched in vehicle-treated *FUS^+/+^* mice and not in ACY-738-treated *FUS^+/+^* mice and nTg controls, demonstrating the effect of HDAC inhibition on lipid metabolism. These LION terms were related to antagonist plasma membrane properties, namely a low bilayer thickness, low transition temperature, and high lateral diffusion. Moreover, LION terms related to fatty acid composition were also enriched. Polyunsaturated fatty acids with 3 or more double bonds and fatty acids with an acyl chain longer than 22 carbons were more abundant. Interestingly, we noted that lipids associated with the mitochondria were more represented in vehicle-treated *FUS^+/+^* mice compared to ACY-738-treated *FUS^+/+^* mice and nTg controls (Figure 4).

Altogether, this lipid ontology enrichment analysis indicates that overexpression of human *FUS* in mice remodeled lipid fatty acid composition by modifying the length and saturation of their acyl chain. Consequently, the perturbation of lipid homeostasis and the alteration of lipids concentration could lead to significant modifications in biophysical membrane properties.

### 2.5. HDAC Inhibition Restores Transcriptional Alterations of Lipid Metabolism Master Transcription Factors and Genes Linked to Glycerophospholipids Synthesis and Remodeling

Finally, we explored a possible direct action of human *FUS* overexpression and ACY-738 treatment on gene expression of master transcription factors involved in lipid metabolism and key enzymes implicated in glycerophospholipids homeostasis. Therefore, we used our previously published transcriptomic data set obtained from spinal cords of 60-day-old vehicle-treated nTg controls and vehicle- or ACY-738-treated *FUS^+/+^* mice [6]. Importantly, mice used to generate the transcriptomic, and lipidomic data sets were generated from different cohorts but housed and treated in the same conditions, allowing us to cross these two data sets.

**Figure 5:**
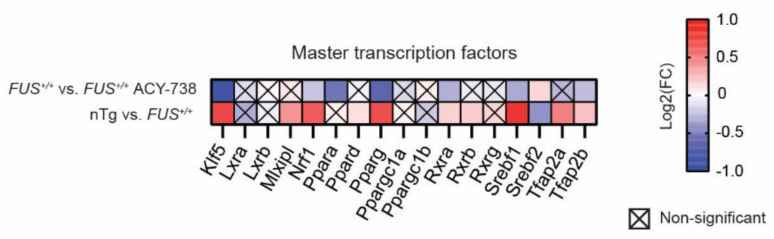
ACY-738 treatment mitigates the activation of master transcription factors involved in lipid metabolism. Heatmap showing color-coded differential expression of significantly down- (blue) or upregulated (red) genes involved in lipid metabolism in the spinal cord of 60-day-old mice. Data are represented as log2 fold change (FC). Crossed squares represent non-significant comparisons. Statistical significance was calculated by 1-way ANOVA. Only genes with a *p* > 0.05, FDR adjusted, were considered as significantly dysregulated. *n* = 6 per group.

Lipid homeostasis involves several metabolic pathways through which lipid species are synthesized or degraded. Master transcription factors regulate the transcriptional activity of the genes implicated in these pathways. Activation of these regulatory proteins leads to a cascade of transcriptional activation, which ultimately conducts to a particular phenotype. We analyzed the expression of the most important transcription factors involved in lipid metabolism (Figure 5). Based on the literature, we selected 17 genes coding for transcription factors [40,41]. Among them, 11 were dysregulated in the spinal cord of 60-day-old *FUS^+/+^* mice compared to nTg controls. Interestingly, we noted the upregulation of the expression of 10 out of 11 of these transcription factors, meaning that human FUS can activate a set of master transcription factors related to lipid metabolism in the mouse spinal cord. Furthermore, ACY-738 treatment significantly induced the downregulation of seven of these transcription factors, namely K*lf5*, *Nrf1*, *Ppara*, *Pparg*, *Rxra*, *Srebf1*, and *Tfap2b*. Furthermore, HDAC inhibition restored the expression of the only downregulated gene in 60-day-old *FUS^+/+^* mice, namely *Srebf2*.

**Figure 6:**
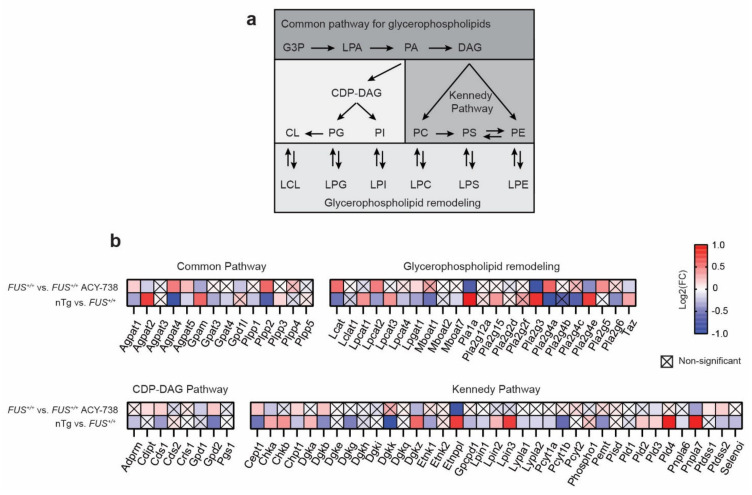
ACY-738 treatment mitigates transcriptomic defects of key enzymes implicated in glycerophospholipid metabolism. (**a**) Schematic of glycerophospholipid metabolism. (**b**) Heatmap showing color-coded differential expression of significantly down- (blue) or upregulated (red) genes involved in glycerophospholipid biosynthesis and remodeling in the spinal cord of 60-day-old mice. Data are represented as log2 fold change (FC). Crossed squares represent non-significant comparisons. Statistical significance was calculated by 1-way ANOVA. Only genes with a *p* > 0.05, FDR adjusted, were considered as significantly dysregulated. *n* = 6 per group.

Glycerophospholipids arise from a common pathway initiated by glycerol 3-phosphate (GP3), which is converted to lysophosphatidic acid (LPA), next converted to phosphatidic acid (PA). Subsequently, PA is metabolized into two types of diacylglycerol (DAG) to incorporate two pathways: the CDP-DAG pathway synthesizing PI, PG, and CL, and the Kennedy pathway producing PC, PE, and PC. Glycerophospholipids contain two acyl chains that can be changed by remodeling through the generation of lysophospholipids, creating the diversity of glycerophospholipid composition (Figure 6a). We detected and selected 82 genes coding for enzymes implicated in glycerophospholipid metabolism based on the KEGG pathway map 00564 [42] (Figure 6b). Among these 82 genes, 56 were found dysregulated by human *FUS* overexpression, of which 21 and 35 genes show increased and reduced expression, respectively. Each pathway of glycerophospholipid biosynthesis was affected, indicating a global effect. Strikingly, we observed that the ACY-738 treatment significantly restored the gene expression alterations of 25 enzymes. Notably, HDAC inhibition targeted genes encoding enzymes that catalyze the conversion of lysophospholipid to phospholipid by adding an acyl group to the glycerol backbone (*Agpat1, 2, 4 and 5, Lcat, Lpcat1, Lpcat2, Taz*), genes encoding phospholipases (*Pla1a, Pla2g3, Pla2g4a, Pla2g4e, Pnpla7*) and genes encoding enzymes implicated in the Kennedy pathway (*Cept1, Chka, Chpt1, Dgkb, Dgkz, Etnppl, Pld3, Ptdss2*).

These transcriptomic data indicate that overexpression of human *FUS* and HDAC inhibition affect the gene expression of key enzymes implicated in the glycerophospholipid metabolism, and particularly the remodeling of subspecies fatty acid composition. Taken together, these data link the perturbation of the lipid homeostasis to primary transcriptional defects in the spinal cord of *FUS^+/+^* mice.

## 3. Discussion

By using a potent HDAC inhibitor, ACY-738, and by combining transcriptomic and lipidomic analysis, our data demonstrated a link between epigenetic modifications and lipid metabolism defects in the spinal cord of a FUS mouse model of ALS. While these two cellular processes appear to be commonly implicated in ALS pathology and actively participate in disease progression [11,25], our results highlight HDAC inhibition as a relevant potential therapeutic strategy to treat this devastating disease.

Previous lipidomic analyses have substantially used mutant SOD1 models. Despite their significant contribution to the understanding of disease-associated mechanisms, mutant SOD1 models showed their limitations when effective therapeutic strategies failed to translate into a benefit for ALS patients. In this study, we used the *FUS^+/+^* mouse model (also known as PrP-hFUS-WT3) developed by Christopher Shaw’s group [21]. Overexpression of human wild-type FUS leads to an accumulation of FUS in the cytoplasm without significantly changing the nucleus-to-cytoplasm ratio [6]. The transgene is consistently expressed from embryonic life throughout adulthood in the nervous system and leads to an aggressive ALS-like phenotype leading to a full hindlimb paralysis with a median survival of 60 days. The first signs of motor impairments arise after 30 days of age, corresponding with the progressive weight loss and decrease in motor performances [6,21]. At the cellular level, these mice suffer from typical neuromuscular junction denervation and motor neuron loss accompanied by a ubiquitin pathology and increased inflammation (astrogliosis and microgliosis) [6,21]. We previously demonstrated that histones hypoacetylation correlates with disease progression since this pathological event is present only in symptomatic 60-day-old *FUS^+/+^* mice [6]. We further established that this pathogenic epigenetic hallmark is due to increased HDAC activity, whereas their mRNA and protein levels remain stable over disease progression [6].

We found that lipid homeostasis was not significantly affected in presymptomatic 25-day-old *FUS^+/+^* mice, unlike what has been shown in mutant SOD1 models [31,32,43]. However, mutant SOD1 mice used in presymptomatic studies are adults (>75-day-old), while in our study, we used juvenile 25-day-old *FUS^+/+^* mice. Our data showed that the lipidome of 25-day-old animals was significantly different compared to 60-day-old animals, with a major proportion of increased individual lipid concentrations. In the mouse spinal cord, puberty is accompanied by a significant increase in axonal extension, synapse remodeling, myelination, and non-neuronal cell numbers [44,45]. Therefore, the lipid composition varies drastically during postnatal mouse development and could hide slight presymptomatic disease-related alterations in *FUS^+/+^* mice. While in the spinal cord of 25-day-old *FUS^+/+^* mice, there is no sign of neurodegeneration nor neuroinflammation. We previously described presymptomatic transcriptional defects related to lipid metabolism [6], suggesting early lipidomic alterations already taking place.

At a late symptomatic stage, *FUS^+/+^* mice recapitulated the lipidomic pathological signature found in the spinal cord of ALS patients and mouse models [26,28,30,31,32,43]. Our lipidomic data confirm the accumulation of specific ceramide species, CE, and the by-product of its generation, LPC. These lipids are found elevated in the spinal cord of ALS patients, *SOD1^G93A,^* and *Sod1^G86R^* models [26,28,30,31,32,43]. LPC is known to be toxic to motor neurons in vitro, while it has been demonstrated that the accumulation of ceramides in the spinal cord exacerbates ALS pathogenesis in a *SOD1^G93A^* mouse model [31,43,46]. We previously showed that genes related to cholesterol metabolism are downregulated in the spinal cord of symptomatic *FUS^+/+^* mice and that ACY-738 partially restored their expression [6]. The lipidomic analysis showed that ACY-738 treatment modestly reduced the accumulation of CE and did not affect LPC concentrations. These results are in line with the hypothesis of a recent study suggesting that CE accumulation is not due to de novo cholesterol synthesis but is instead driven by the transfer of acyl groups from PC to cholesterol [30]. Although drugs designed to specifically target sphingolipids homeostasis have beneficial effects in preclinical models [31,37,43], we showed here that HDAC inhibition had almost no impact on the concentration of individual lipid species and failed to diminish the increased ceramide concentrations. Therefore, the beneficial effects observed in our model are not attributed to the modulation of sphingolipids.

On the contrary, our study reveals that glycerophospholipids alterations are the prime targets of ACY-738 therapy. Glycerophospholipids were among the most represented discriminant lipids distinguishing *FUS^+/+^* mice from nTg controls, confirming the important rearrangements of these lipid classes found in ALS patients and SOD1 models [24,26,27,28,29,32]. The concentrations of these glycerophospholipids and, more critically, the molar ratio between PC and PE are critical parameters of cellular health. Perturbed PC/PE ratio leads to impairments of plasma membranes, lipid droplets, lipoproteins, ER, and mitochondria functions [38]. ACY-738 targeted specific PC and PE species to restore similar concentrations to nTg controls and therefore reinstated a homeostatic PC/PE ratio. Glycerophospholipids are the building blocks of plasma and organelle membranes. Enrichment or diminishment of specific species and the variation of their fatty acid composition influence the membrane biophysical properties, such as curvature, thickness, permeability, and fluidity [47]. Our lipid ontology enrichment analysis suggests that human *FUS* overexpression in mice impacts membrane properties by enriching lipids containing polyunsaturated fatty acids and fatty acids with a long acyl chain. It has been suggested that changes in polyunsaturated fatty acids reflect altered susceptibility to lipid peroxidation and/or the presence of an inflammatory process [26]. The remodeling of glycerophospholipid fatty acid composition was confirmed by the increased concentrations of several lysophospholipid species (LPS, LPC, LPE O), which serve as intermediate precursors to incorporate fatty acids [48]. Interestingly, ACY-738 treatment could reverse part of the remodeling of fatty acids and the modification of membranes properties. CL is a specific glycerophospholipid class exclusively located in inner mitochondrial membranes, promoting the formation of highly curved regions [49]. CL plays a crucial role in mitochondrial function and is involved in the formation and maintenance of protein-protein and protein-membrane interactions, ATP production, bioenergetics, mitophagy, and apoptotic signaling pathways [49]. We observed a decreased concentration of CL in *FUS^+/+^* mice, suggesting dysfunction in mitochondrial activity [28]. Restoration of CL levels by ACY-738 indicates that HDAC inhibition beneficial effects could be in part mediated by an increased mitochondrial capacity and the amelioration of motor neuron bioenergetic.

Furthermore, we found that genes coding for enzymes contributing to glycerophospholipid metabolism is dysregulated in *FUS^+/+^* mice and corrected by ACY-738 treatment. Glycerophospholipid biosynthesis predominantly occurs in the cytosolic part of the endoplasmic reticulum (ER) and requires the close interaction of mitochondria and ER membranes at specific sites called mitochondria-associated ER membranes (MAMs) where biosynthetic enzymes are concentrated [50]. MAMs are involved in lipid transport, glycerophospholipid, and cholesterol synthesis, calcium homeostasis, ER stress, ROS generation, autophagy, and mitochondria dynamics [51]. MAMs defects have been repeatedly associated with ALS [52]. Indeed, mutations in SIGMAR1 and VAPB, transmembrane proteins involved in MAMs functions, provided a direct link between MAMs defects and neurodegeneration in ALS [53,54,55]. Moreover, recent studies also found the involvement of SOD1, FUS, and TDP-43 in these alterations [53,54,56]. *FUS^+/+^* mice are characterized by a decreased number of contact sites between the mitochondria and ER [56]. Moreover, we previously demonstrated in vitro, on motor neurons derived from *FUS*-ALS patients, that ACY-738 treatment ameliorated MAMs disruption and subsequently restored the extracellular PC decreased concentrations [12]. Therefore, we can hypothesize that ACY-738 mediates its effects by restoring MAMs normal functions while ameliorating lipid homeostasis, particularly glycerophospholipids.

Two questions arise from our bulk untargeted lipidomic analysis, which the answers will be essential to develop highly selective therapeutic strategies. (1) Which cell types are involved in the lipidomic defects? (2) Which HDAC family members targeted by ACY-738 are implicated in the modulation of lipid metabolism?

When studying lipids in ALS, deciphering the contribution of individual cell types to the pathological processes remains challenging [25]. Moreover, the observed phenotype could be directly related to neuron or glial cells biology/health or related to their changing proportions over disease progression. While the ACY-738 therapy rescued some lipidomic alterations in the spinal cord of symptomatic 60-day-old *FUS^+/+^* mice, the treatment did not rescue the 60% spinal motor neuron loss and did not affect the development of astrogliosis and microgliosis [6]. Therefore, these results suggest that the lipid metabolism defects are more likely due to cellular pathophysiological processes than a change in the cellular proportions. Furthermore, investigation of the expression levels of lipid-related genes provided us further insights into the possible molecular mechanisms by which ACY-738 is modulating lipid concentrations. Master transcription factors adjust lipid homeostasis by activating cascades of transcriptional activation and therefore regulating the expression of multiple genes. Principal master transcription factors involved in lipid metabolism were activated in *FUS^+/+^* mice and downregulated by ACY-738 treatment, indicating an upstream effect of HDAC inhibition over lipid metabolism. Interestingly, we found that *Srebf2*, a transcription factor regulating specifically cholesterol metabolism, was downregulated in *FUS^+/+^* mice and upregulated by ACY-738. A recent study showed that FUS plays a critical in oligodendrocytes by regulating myelination through cholesterol metabolism [57]. Moreover, genetic deletion of *TDP-43* in mouse oligodendrocytes leads to decreased expression of *Srebf2*, disrupted cholesterol metabolism, and a severe demyelination phenotype [58]. Combined with our results, these studies point to a potential effect of human FUS on myelin or oligodendrocytes development/differentiation, which could explain the lack of alteration in 25-day-old animals and their existence in 60-day-old animals when the developmental myelination period is over in the mouse spinal cord [44,45]. Moreover, cholesterol-related genes are already downregulated in 25-day-old *FUS^+/+^* mice, suggesting an early involvement [6]. Previous lipidomic studies in the spinal cord of *SOD1* models have pointed to specific alterations in neurons and astrocytes. Analysis of the nuclear lipidome of *SOD1* motor neurons showed defects mostly in glycerophospholipids, diacylglycerol, and triacylglycerides [59], whereas a bulk lipidomic analysis suggested the accumulation of CE in astrocytes lipid droplets [28]. Although, accumulation of lipid droplets has also been observed in motor neurons and more recently in microglia of aged brains [59,60]. Intriguingly, the role of microglia in lipid homeostasis has never been explored in ALS. Altogether, these studies suggest that the lipid defects observed in *FUS^+/+^* mice could arise from different cell types. In vitro studies will help us answer the question of the contribution of individual cell types, even if these systems lack the complete environment and the crosstalk between neurons and glial cells necessary for lipid homeostasis. Moreover, the development of in vivo single-cell lipidomic will revolutionize the field in the near future [61].

The pathophysiological relationship between HDACs and FUS is still not clearly understood. Dysregulated HDACs are a common feature of FUS-ALS models and patients [62], which has been associated with impaired DNA damage and stress response [16,63], chromatin remodeling [64], and histone hypoacetylation [6]. Interestingly, recent studies showed that FUS contains three potential acetylation sites [65], which could play an important role in FUS pathology since combined inhibition of HDAC1 and HDAC3 enhanced nuclear localization of FUS [16]. However, we previously showed that HDAC inhibition with ACY-738 reduced pathological cytoplasmic FUS accumulation but failed to increase FUS nuclear localization, suggesting that the beneficial effects of HDAC inhibition are independent of FUS nuclear localization and its transcriptional activity [6]. Furthermore, we established that the beneficial effects of ACY-738 treatment in *FUS^+/+^* mice are not primarily mediated by HDAC6 inhibition [6], suggesting that HDAC1, HDAC2, or HDAC3 are involved. These nuclear class I HDAC members have been recently linked to lipid metabolism. In vitro, HDAC2 activity has been shown to reduce cardiolipin levels through the degradation of ALCAT1, leading to mitochondrial fragmentation and malfunction, whereas inhibition of HDAC2 activity increased cardiolipin levels [66]. Furthermore, HDAC3 genetic deletion also increased cardiolipin levels in skeletal muscles, plus modified free fatty acid concentrations and remodeled specific ceramides molecular species [67]. Thus, HDAC2 and HDAC3 inhibition could explain the restoration of cardiolipin concentrations in *FUS^+/+^* mice. Interestingly, HDAC1, which directly interacts with FUS [63,68], regulates lipid and fatty acid metabolic processes in enteroids [69], while in the liver and adipose tissues, HDAC3 controls lipid and cholesterol synthesis and their storage [70,71,72]. Recent studies found that HDAC3 adjusts skeletal muscles fuel metabolism, and HDAC3 deletion has been shown to reduce glucose use, favoring lipid oxidation [73,74]. However, a comparable metabolic switch is known to drive disease onset in skeletal muscles of *Sod1^G86R^* mice and would therefore exacerbate the disease progression [75]. Altogether, these studies and our highlight the critical role of class I HDAC in lipid metabolism, not only in the central nervous system but also in peripheral organs. Therefore, we can’t exclude an indirect effect of HDAC inhibition from the periphery to the spinal cord.

Recent studies demonstrated the potential protective effect and benefit of lipids modifications in ALS [25]. The modulation of sphingolipids using different pharmacological agents has been shown to drive improvements in motor performances, protection of neuromuscular junctions, and prolong survival of SOD1 models [26,31,37]. Our lipidomic approach demonstrated that HDAC inhibition is a novel tool to modulate lipid metabolism in the central nervous system. Modification of glycerophospholipid metabolism by ACY-738 is accompanied by the amelioration of motor performances, the integrity of neuromuscular junctions, and significantly increased the survival of FUS mice. Moreover, a recent study showed that ALS patients are characterized by dysregulated glycerophospholipid blood levels at an early stage of the disease and that specific glycerophospholipids species correlate with disease progression [24,76]. Therefore, these particular lipid alterations could serve as effective biomarkers, prognosis markers, and potent indicators for the effectiveness of new HDAC inhibitors, adding a stepping stone for the rapid development of innovative treatments.

Altogether, this study confirms the therapeutic potential of HDAC inhibition for ALS and the necessity to investigate the underlying molecular mechanisms in more detail.

## 4. Materials and Methods

### 4.1. Animals

This animal study was performed by authorized investigators and approved by the local ethical committee of the KU Leuven, Leuven, Belgium (P055-2014) and comply with the current laws of Belgium. Mice were housed at the KU Leuven animal facilities, according to the in-house guidelines. Mice were kept on a 12 h light-dark cycle at a temperature of 20 °C with food and water ab libitum. Transgenic *FUS^+/−^* breeding mice (stock no. 017916, also known as PrP-hFUS-WT3) were purchased from The Jackson Laboratory. Mouse genotyping was performed as previously described [6]. Briefly, DNA was isolated and amplified from ear biopsies. The human *FUS* transgene was quantified by qPCR with a FAM-labeled probe (forward and reverse primers 5′-CAGCAAAGCTATGGACAGC-3′3′; 5′GTCTTGATTGCCATAACCGC-3′ and Taqman prob 5′-AGCAGAACCAGTACAACAGCAGCA-3′3′). β-actin was used as a reference gene (forward and reverse primers 5′-CCCTACAGTGCTGTGGGTTT-3′3′; 5′-GACATGCAAGGAGTGCAAGA-3′3′). Non-transgenic (nTg) and transgenic mice homozygously overexpressing human *FUS* (*FUS^+/+^*) were randomly selected and assigned to an experimental group: non-treated and sacrificed at 25 days; vehicle- or ACY-738-treated and sacrificed at 60 days (*n* = 3 per group). Both males and females were used as we previously demonstrated no sex effect on the ALS phenotype and ACY-738 treatment response [6]. Littermates were used as controls. Drug administration was performed as previously described [6]. Crushed Teklad LM-485 sterilized rodent diet (Envigo, Cambridgeshire, U.K.) mixed with water was used as a vehicle and provided *ab libitum* in the home cage from the age of 28 to 30 days. Diet for the ACY-738-treated group contained 100 mg/kg of ACY-738 powder (Regenacy Pharmaceuticals Inc., Waltham, MA, USA). Mice were daily followed for well-being, weight loss, and disease progression. To monitor further the efficiency of ACY-738 therapy, *FUS^+/+^* mice performed twice a week the grip strength test and the amplitude of the compound muscle action potential was measured once a week, as previously described [6]. For spinal cord collection, mice were euthanized with CO_2_ followed by cervical dislocation. Spinal cords were rapidly dissected and snap-frozen in liquid nitrogen. Samples were stored at −80 °C and shipped on dry ice to Lipotype GmbH (Dresden, Germany) to perform untargeted lipidomic profiling.

### 4.2. Lipid Nomenclature

Classification and nomenclature of lipid follow the LIPID MAPS recommendations [77]. Lipid classes are described in Supplementary Table S1. Lipid species are annotated according to their molecular composition as <Name of the lipid class> <sum of the carbon atoms in the hydrocarbon moiety>:<sum of the double bonds in the hydrocarbon moiety>. For example, SM 42:1 denotes a sphingomyelin species- with a total of 42 carbon atoms and 1 double bond. Fragmentation of the lipid molecules can deliver subspecies information on the exact identity of their acyl moieties and their *sn*-position (MSMS mode only, see Supplementary Table S1). For example, PE 18:0_20:4 denotes a phosphoethanolamine species with stearic acid (18:0) and arachidonic acid (20:4) fatty acids, for which the exact *sn*-position cannot de discriminated (underline “_” separating the acyl chains). On the contrary, and only for PC O- and PE O- species, slash “/” separating signifies that the *sn*-position can be resolved. In the case of ceramides, the first entity denotes a sphingoid base and the second a fatty acid. Identical ceramide species were differentiated by their number of hydroxyl groups, indicated after a semicolon. For example, Cer 36:1; 2 denotes a ceramide with 2 hydroxyl groups.

### 4.3. Lipid Extraction for Mass Spectrometry Lipidomics

Mass spectrometry-based lipid analysis was performed by Lipotype GmbH (Dresden, Germany) as described [78]. Lipids were extracted using a two-step chloroform/methanol procedure [79]. Samples were spiked with internal lipid standard mixture containing: cardiolipin 16:1/15:0/15:0/15:0 (CL), ceramide 18:1; 2/17:0 (Cer), diacylglycerol 17:0/17:0 (DAG), hexosylceramide 18:1; 2/12:0 (HexCer), lyso-phosphatidate 17:0 (LPA), lyso-phosphatidylcholine 12:0 (LPC), lyso-phosphatidylethanolamine 17:1 (LPE), lyso-phosphatidylglycerol 17:1 (LPG), lyso-phosphatidylinositol 17:1 (LPI), lyso-phosphatidylserine 17:1 (LPS), phosphatidate 17:0/17:0 (PA), phosphatidylcholine 17:0/17:0 (PC), phosphatidylethanolamine 17:0/17:0 (PE), phosphatidylglycerol 17:0/17:0 (PG), phosphatidylinositol 16:0/16:0 (PI), phosphatidylserine 17:0/17:0 (PS), cholesterol ester 20:0 (CE), sphingomyelin 18:1;2/12:0; 0 (SM), triacylglycerol 17:0/17:0/17:0 (TAG). After extraction, the organic phase was transferred to an infusion plate and dried in a speed vacuum concentrator. First step dry extract was re-suspended in 7.5 mM ammonium acetate in chloroform/methanol/propanol (1:2:4, V:V:V) and second step dry extract in 33% ethanol solution of methylamine in chloroform/methanol (0.003:5:1; V:V:V). All liquid handling steps were performed using Hamilton Robotics STARlet robotic platform with the Anti Droplet Control feature for organic solvents pipetting.

### 4.4. MS Data Acquisition

Samples were analyzed by direct infusion on a QExactive mass spectrometer (Thermo Scientific, Waltham, MA, USA) equipped with a TriVersa NanoMate ion source (Advion Biosciences, Ithaca, NY, USA). Samples were analyzed in both positive and negative ion modes with a resolution of Rm/z = 200 = 280,000 for MS and Rm/z = 200 = 17,500 for MSMS experiments in a single acquisition. MSMS was triggered by an inclusion list encompassing corresponding MS mass ranges scanned in 1 Da increments [80]. Both MS and MSMS data were combined to monitor CE, DAG, and TAG ions as ammonium adducts; PC, PC O-, as acetate adducts, and CL, PA, PE, PE O-, PG, PI, and PS as deprotonated anions. MS only was used to monitor LPA, LPE, LPE O-, LPI, and LPS as deprotonated anions; Cer, HexCer, SM, LPC, and LPC O- as acetate adducts.

### 4.5. Lipidomic Data Processing

Data were analyzed with in-house developed lipid identification software based on LipidXplorer [81,82]. Data post-processing and normalization were performed using an in-house developed data management system. Only lipid identifications with a signal-to-noise ratio > 5 and a signal intensity 5-fold higher than in corresponding blank samples were considered for further data analysis. The amounts in pmoles of individual lipid molecules of a given lipid class were summed to yield the total amount of the lipid class. The amounts of the lipid classes were normalized to the total lipid amount yielding mol% per total lipids.

### 4.6. Statistical Analysis of Lipidomic Data

Statistical analysis was performed on normalized data (mol%) with GraphPad Prism 9.0 (GraphPad Software Inc., San Diego, CA, USA) and MetaboAnalyst 5.0 (online software, www.metaboanalyst.ca, accessed on 12 April 2021) [83]. First, data were imputed to remove missing values. Lipid species with >50% missing values were removed, and the remaining missing values were replaced by LoDs (1/5 of the minimum positive value of each lipid species). To perform univariate and multivariate analyses in MetaboAnalyst, data were log2 transformed and auto-scaled (mean-centered and divided by the standard deviation of each lipid species). To obtain the overall picture of the data set, all groups were compared by principal component analysis (PCA) and hierarchical clustering heatmaps (distance measure: Euclidean, clustering algorithm: ward, standardization: auto-scale features). Sixty-day-old groups were compared by one-way ANOVA followed by Tukey’s post-hoc test (*p* < 0.05; FDR adjusted). Heatmap was used to display the TOP50 most altered lipid species. To analyze which lipid species contributes to the differences between groups, partial least squares discriminant analysis (PLS-DA) was employed. The TOP15 lipid species that are responsible for the discrimination of the phenotypes are plotted using variable importance of projection (VIP). A volcano plot analysis was conducted to identify the overall altered lipid profile. The fold change was set at FC > 1.2 and the *p*-value < 0.05 (*t*-test, FDR adjusted). To classify significantly dysregulated lipids, an enrichment analysis was conducted using the web application (LION/web) [39]. Enrichment analysis of lipid ontology terms (LION) was performed on normalized data from MetaboAnalyst and subjected to the LION-PCA heatmap module. Each LION term represents a group of lipid species that share common properties, e.g., biophysical properties, fatty acid structure, organelle association, etc. The most fluctuating LION terms were selected by assessing enrichment over a set number of principal components, as previously described [84]. Principal components with a cumulative variance between 80% and 90% were displayed on the heatmap. To calculate the concentrations of lipid species per class or lipids with similar acyl chain properties, individual lipid species concentrations from normalized data were summed. For comparison, one-way ANOVA followed by Tukey’s post-hoc test was performed. Results are presented as the mean ± standard error of the mean (SEM).

### 4.7. Transcriptomic Data Acquisition and Statistical Analysis

Glycerophospholipid metabolism-related gene expressions were analyzed with the previous published transcriptomic data set [6]. In this former study, transgenic *FUS^+/+^* mice were housed and treated in the same condition, and spinal cord tissues were collected at 60 days with the same protocol, as defined above. Rossaert and collaborators evaluated the differential expression of genes by RNA sequencing with an edgeR analysis. Multiple testing correction was performed using FDR Benjamini–Hochberg correction. Heatmap was used to display the log2 fold change as a representation of the variation of their expression.

## Data Availability

Lipidomic data that support the findings of this study are available within the Supplementary Data File. Transcriptomic data previously published are available from the corresponding author on reasonable request.

## Supplementary Materials

The following are available online at https://www.mdpi.com/article/10.3390/ijms222011224/s1.

## Abbreviations

ALS	Amyotrophic lateral sclerosis
C9ORF72	Chromosome 9 open reading frame 72
CE	Cholesterol esters
Cer	Ceramide
CL	Cardiolipin
DAG	Diacylglycerol
ER	Endoplasmic reticulum
FA	Fatty acids
FC	Fold change
FUS	Fused in sarcoma
GP3	Glycerol 3-phosphate
HCA	Hierarchical clustering analysis
HDAC	Histone deacetylase
HexCer	Hexocylceramide
LION	Lipid ontology
LPA	Lyso-phosphatidate
LPC/LPC O-	Lyso-phosphatidylcholine (-ether)
LPE/LPE O-	Lyso-phosphatidylethanolamine (-ether)
LPG	Lyso-phosphatidylglycerol
LPI	Lyso-phosphatidylinositol
LPS	Lyso-phosphatidylserine
MAMs	Mitochondria-associated ER membranes
nTg	Non-transgenic
PA	Phosphatidate
PCA	Principal component analysis
PC/PC O-	Phosphatidylcholine (-ether)
PE/PE O-	Phosphatidylethanolamine (-ether)
PG	Phosphatidylglycerol
PI	Phosphatidylinositol
PLS-DA	Partial least squares discriminant analysis
PrP	Prion protein promotor
PS	Phosphatidylserine
SEM	Standard error of the mean
SM	Sphingomyelin
SOD1	Superoxide dismutase 1
TAG	Triacylglycerol
TARDBP	TAR DNA binding protein 43
VIP	Variable importance in projection
