# Supplementary material for: Histone Deacetylase Inhibition Regulates Lipid Homeostasis in a Mouse Model of Amyotrophic Lateral Sclerosis
Source: Int J Mol Sci. 2021 Oct 18;22(20):11224. doi: 10.3390/ijms222011224 (PMC8541517; doi:10.3390/ijms222011224)
Supplement: Supplementary file 1 [file ijms-22-11224-s001.zip › Burg_2021_Supplementary_IJMS.pdf]

Histone deacetylase inhibition regulates lipid homeostasis in a mouse model of amyotrophic lateral sclerosis

Thibaut Burg [1,2], Elisabeth Rossaert [1,2] Matthieu Moisse [1,2], Philip Van Damme [1,2,3] and Ludo Van Den Bosch [1,2,*]

[1] Department of Neurosciences, Experimental Neurology, Leuven Brain Institute, KU Leuven – University of Leuven, Leuven, Belgium; thibaut.burg@kuleuven.be (T.B.); elisabeth.rossaert@kuleuven.be (E.R.); matthieu.moisse@genadvice.com (M.M.); philip.vandamme@kuleuven.be (P.V.D.).

[2] Center for Brain & Disease Research, Laboratory of Neurobiology, VIB, Leuven, Belgium.

[3] University Hospitals Leuven, Department of Neurology, Leuven, Belgium.

* Correspondence: ludovandenbosch@kuleuven.be (L.V.D.B.).

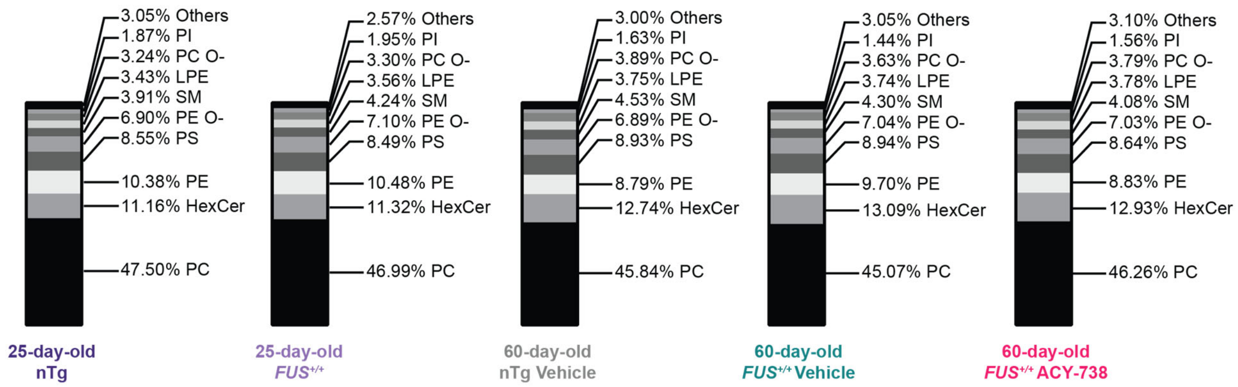

Supplementary Figure S1. Proportion of the identified lipid classes in the mouse spinal cord. Average distribution in percentage of the 9 most abundant lipid classes in the spinal cord of 25-day-old non-treated nTg controls and *FUS*$^{+/+}$ mice, 60-day-old vehicle-treated nTg controls and vehicle- or ACY-738-treated *FUS*$^{+/+}$ mice. n=3 per group. 'Others' is the sum of the remaining lipid classes with proportions below 1%.

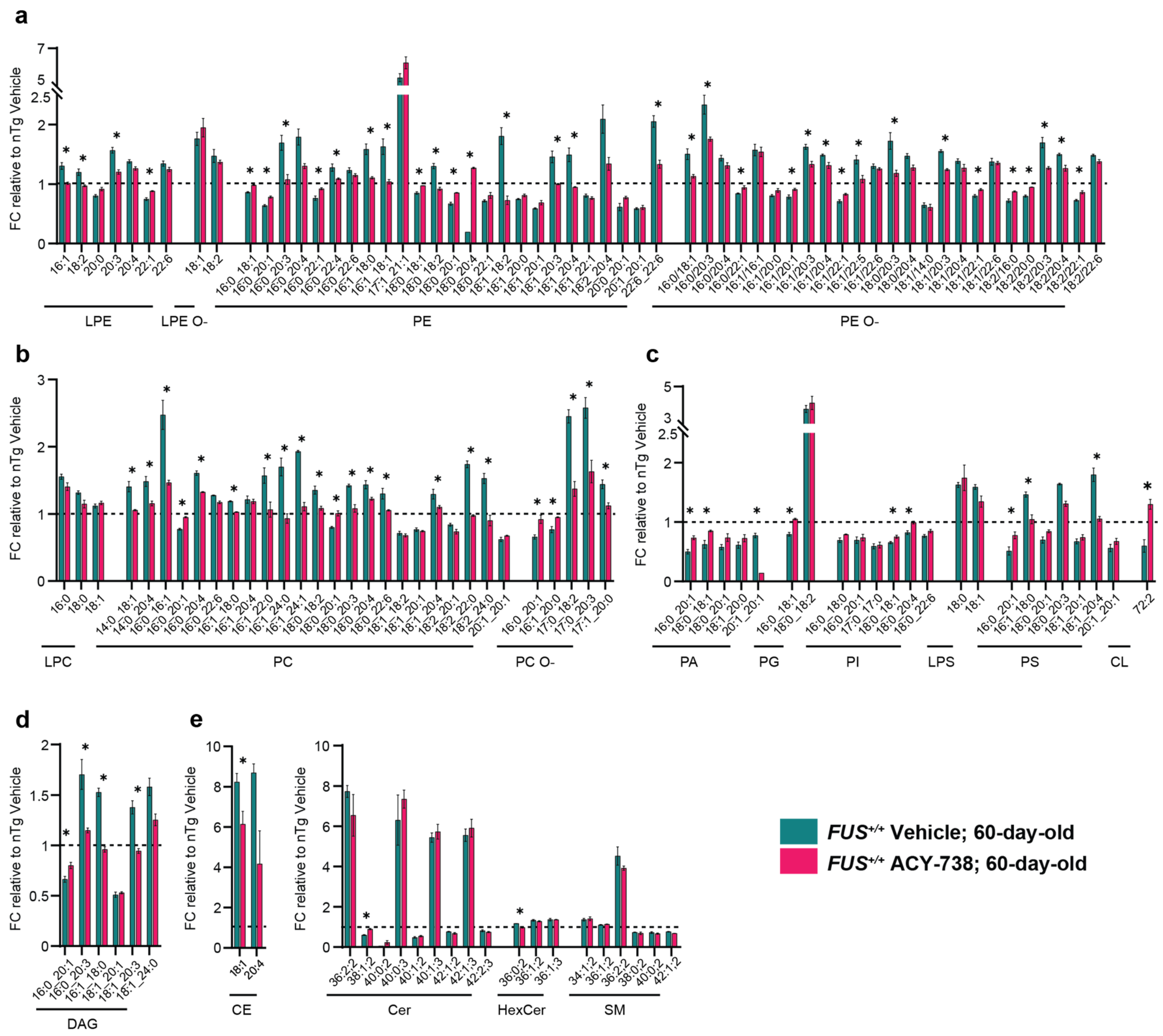

Supplementary Figure S2. Normalized concentrations of significantly dysregulated lipids species relative to 60-day-old vehicle-treated nTg. Lipid species concentrations of 60-day-old vehicle- or ACY-738-treated *FUS*$^{+/+}$ mice are represented as the fold change (FC) relative to 60-day-old vehicle-treated nTg controls. A value of 1 indicates no change in concentration. (**a-e**) Only lipid species that are significantly dysregulated in 60-day-old vehicle-treated *FUS*$^{+/+}$ mice compare to vehicle-treated nTg controls are represented. * indicates a significant difference of concentration between vehicle- and ACY-738-treated *FUS*$^{+/+}$ mice. Glycerophospholipids with an ethanolamine or choline headgroup are displayed in (a) and (b), respectively. Other glycerophospholipids are displayed in (c). Diacylglycerols are represented in (d). Cholesterol esters (CE) and sphingolipids are displayed in (e) and (f), respectively. Data are presented as mean ± standard error of the mean (SEM), n=3. Statistical significance was calculated by 1-way ANOVA, followed by Tukey's post hoc test. *p<0.05, FDR corrected.

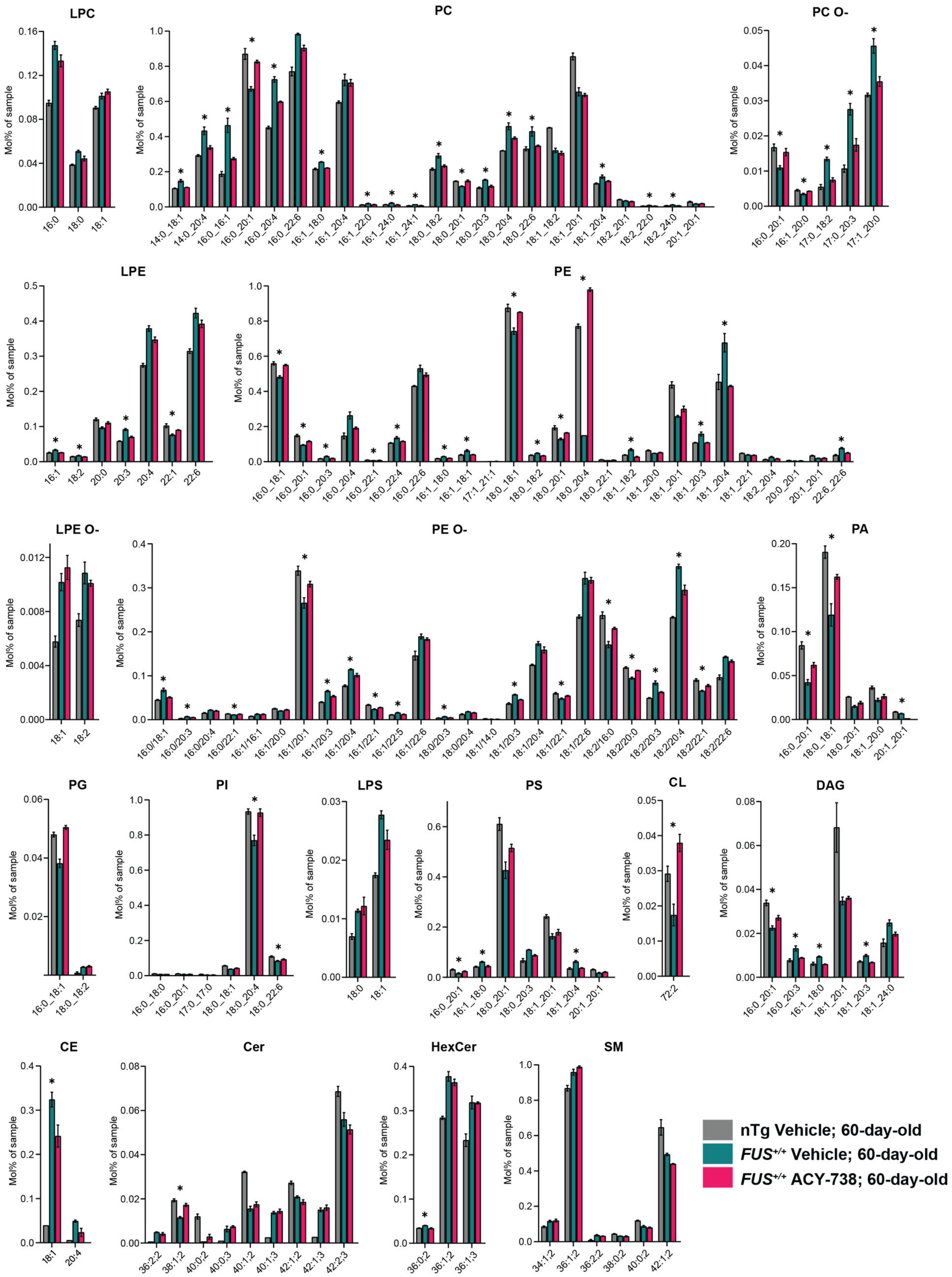

Supplementary Figure S3. Concentrations of significantly dysregulated lipids species in mol% of 60-day-old mice. Only

lipid species that are significantly dysregulated in 60-day-old vehicle-treated *FUS*$^{+/+}$ mice compare to nTg controls are represented. * indicates a significant difference of concentration between vehicle- and ACY-738-treated *FUS*$^{+/+}$mice. Data are in percentage of moles of sample, presented as mean ± standard error of the mean (SEM). Statistical significance was calculated by 1-way ANOVA, followed by Tukey's post hoc test. *$p<0.05$, FDR adjusted.
